# Successful Treatment of Refractory Cutaneous Manifestations of Dermatomyositis With Intravenous Immunoglobulin: A Case Report

**DOI:** 10.7759/cureus.68518

**Published:** 2024-09-03

**Authors:** Yousif B Aldoseri, Sahar Saad, Khalid B Aldoseri

**Affiliations:** 1 Internal Medicine, King Hamad University Hospital, Busaiteen, BHR; 2 Rheumatology, King Hamad University Hospital, Busaiteen, BHR

**Keywords:** steroid refractory, skin lesions, refractory disease, intravenous immunoglobulins (ivig), adult-onset dermatomyositis

## Abstract

Dermatomyositis is a connective tissue disorder with dermatological and extracutaneous manifestations. Multiple treatment modalities have been used to treat dermatomyositis, and there have been cases with resistant disease refractory to conventional therapies. Corticosteroids are usually used to achieve remission, and from then, steroid-sparing agents such as azathioprine are used to maintain remission. However, in this report, we present a case of dermatomyositis with refractory cutaneous manifestation. The patient’s muscular pain had subsided with the use of corticosteroids, but the dermatological lesions did not respond to multiple treatment modalities, responding only to intravenous immunoglobulins (IVIG).

## Introduction

Dermatomyositis is a connective tissue disorder that presents with cutaneous and extracutaneous manifestations. It affects the skin and muscles, and the disease may involve other organs such as the lungs. Dermatomyositis is also known to be associated with malignancies [1]. Different treatment modalities have been used to treat dermatomyositis. Initial treatment regimens consist of using corticosteroids. However, long-term use of corticosteroids can lead to adverse events [2]; thus, steroid-sparing agents can be used, for example azathioprine, in combination with hydroxychloroquine [3]. Case studies have also shown that methotrexate in combination with prednisolone can be used in the management of dermatomyositis and achieve remission [4]. Rituximab has been previously explored as a treatment option for treating patients with refractory skin and muscle diseases, but it may also fail to achieve the desired therapeutic effect [5]. Intravenous immunoglobulins (IVIG) have also been proposed as a treatment modality that can be used to treat refractory disease, which is explored in this case report.

## Case presentation

**Figure 1:**
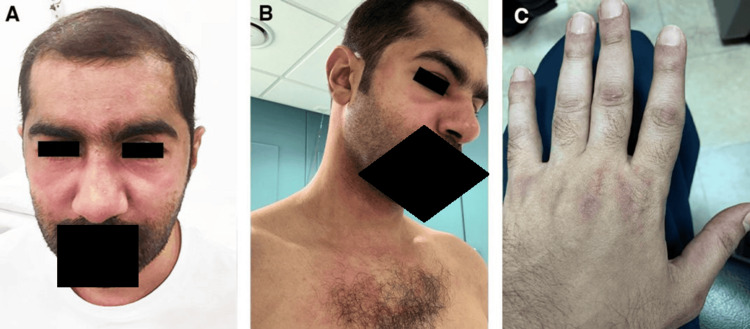
Initial skin manifestations A: Heliotrope rash; B: Shawl sign; C: Gottron papules

We present a 42-year-old man with no comorbidities who was referred to the rheumatology clinic to assess his joint pain and muscle aches of two weeks’ duration with difficulty in swallowing, which the patient described was moderate in severity. The patient had rashes affecting the face, eyelids (heliotrope rash) (Figure 1A), scalp, upper extremities, lower extremities, and trunk for a few months (Figure 1B). The patient also had papules on both extensor surfaces of his hands, mainly over his knuckles (Gottron papules) (Figure 1C). The skin lesions were extensive and debilitating to the patient.

He had been prescribed prednisolone 20 mg once daily by a physician at the time of presentation for the skin lesions, leading to slight improvement. He had a positive family history as his mother has rheumatoid arthritis.

Laboratory investigations sent on the 5th of October 2020 revealed a negative autoimmune profile, including anti-Mi-2 and anti-Jo1 antibodies. Creatine kinase (CK) and CKMB and aldolase levels were high (Table 1). Urine analysis revealed proteinuria of 50 mg/dL initially; however, this subsided one month later. Vitamin D and B12 levels were low and were treated with intramuscular injections.

**Table 1:** Initial investigations

Investigation	Result	Reference Range
Autoimmune profile	Negative	
Anti-Jo1	Negative	
Anti-Mi-2	Negative	
Creatine kinase	581 U/L	35-232 U/L
CKMB	7.84 ng/mL	0-5 ng/mL
Aldolase	12.2 U/L	< 7.6 U/L
Anti-TIF1-gamma antibody levels	Positive	

A skin punch biopsy showed an atrophic epidermis, with prominent vacuolar interface change, with spare perivascular lymphocytic infiltrate with increased dermal mucin, with muscle fibers showing focal atrophy with fragmentation and necrosis. Treatment was initiated by administering three doses of pulse steroid methylprednisolone 1 gm over three days. After receiving pulsed steroids, the patient was started on azathioprine 50 mg twice daily, prednisolone 40 mg once daily, and hydroxychloroquine 300 mg once daily. One month after the initiation of treatment, on the 4th of November 2020, the patient’s CK level dropped to 366 U/L and level of CKMB to 4.35 ng/mL. Muscle pains had improved; however, the skin rashes had persisted. Topical calcineurin inhibitors and corticosteroids were tried, but no improvement was observed. The patient had then developed muscle weakness due to steroid-induced myopathy, and so the dose of prednisolone was tapered gradually to 20 mg once daily. The dose of azathioprine was increased to 50 mg three times daily.

The patient then developed a febrile illness and was admitted to the hospital on the 29th of November 2020. The patient was found to have a cytomegalovirus infection and was treated with antiviral therapy and ibuprofen in view of his paracetamol allergy. Azathioprine was held during admission. Upon discharge, the medications were resumed, and the patient had an increase in the size of the rash on his face, and the dose of prednisolone was increased from 30 mg once daily to 40 mg once daily. The patient also developed palpitations, which were suspected to be due to azathioprine, and so the dose was reduced to 50 mg twice daily. The palpitations have subsided after reducing the dose of azathioprine.

On the 7th of December 2020, the level of CK had decreased then to 70.9 U/L, and the level of CKMB decreased to 1.85 ng/mL, with gradual resolution of muscle weakness. The patient did not experience further muscle pain or muscle weakness afterward, and the difficulty in swallowing had resolved as well. The patient had a significant hair fall with the use of azathioprine, and so it was discontinued. After discontinuation, the hair loss had subsided. Hydroxychloroquine was also discontinued as joint pains have resolved. Alternative treatment modalities were considered, and so rituximab was started for the patient. The patient received two doses of rituximab, on the 25th of May 2021, and on the 9th of June 2021, at a dose of 1 gm. However, no improvement was observed, and it was discontinued.

**Figure 2:**
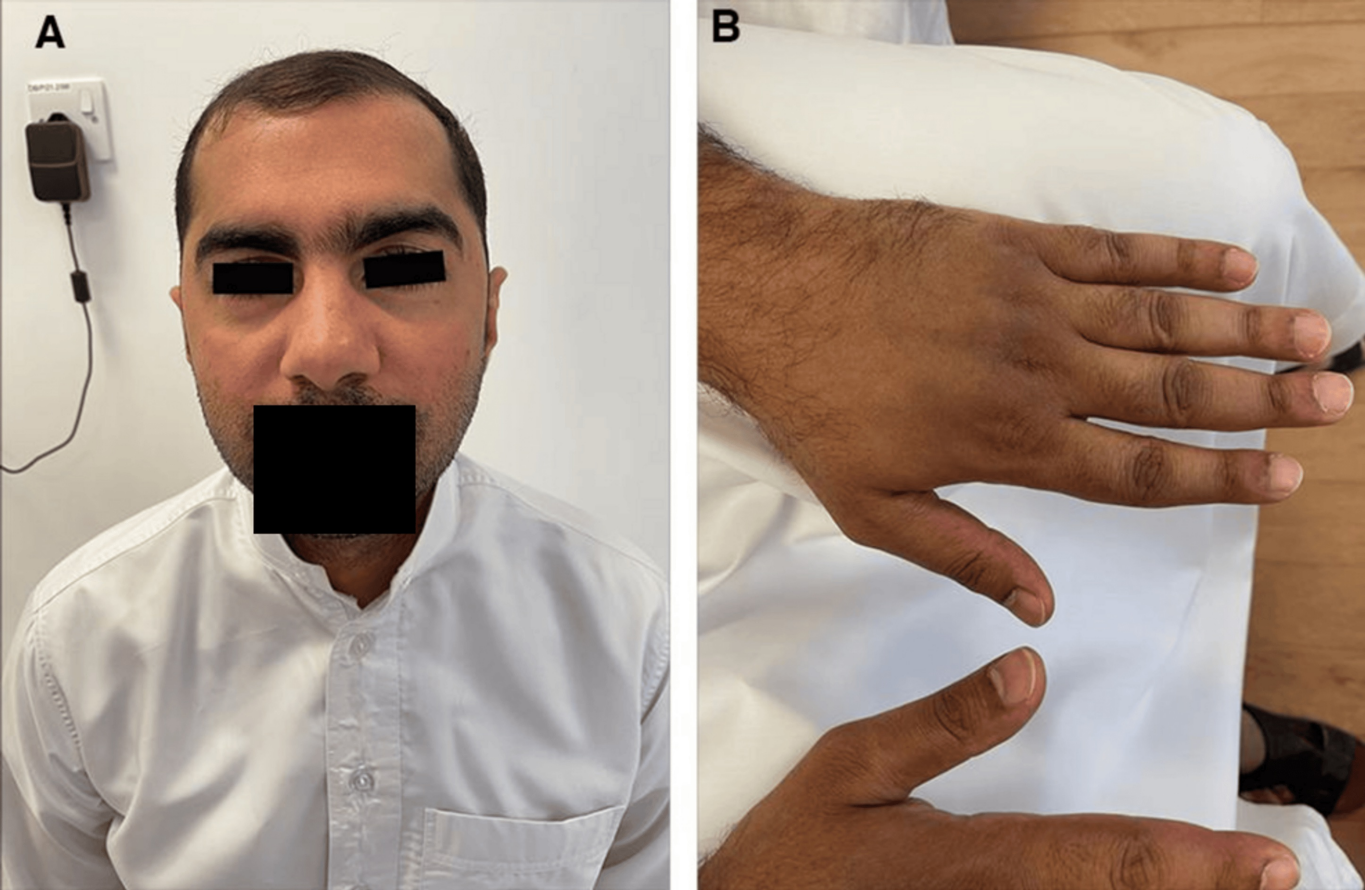
Skin manifestations after treatment with intravenous immunoglobulins (IVIG) A: Resolution of heliotrope rash; B: Resolution of Gottron papules

IVIG was then started for the patient at a four-weekly interval at a dose of 1 gm/kg/day on two consecutive days. Upon initiation of treatment with IVIG, the skin lesions had good improvement, and the patient remained on this treatment regimen. Prednisolone was also tapered gradually to a dose of 7.5 mg once daily, and methotrexate was started at a dose of 10 mg once weekly. The interval between doses was attempted to be increased to a six-weekly interval. However, this caused a recurrence of skin rashes, and so the dosing interval was changed back to being every four weeks (Figures 2A-2B).

In view of the association between dermatomyositis and possible malignancies, further investigations were sought. Anti-TIF1-gamma antibody levels were positive, which is highly associated with malignant tumors associated with dermatomyositis [6]. PET scans were done annually as well, for a total of three times, and, currently, no malignancy has been detected.

The patient had also developed an oozing skin lesion over his left lateral malleolus, which had resolved without surgical intervention. A sample was taken from the fluid, which was found to be sterile.

## Discussion

The patient had been tried on multiple treatment modalities. With the use of corticosteroids, the patient had modest improvement in cutaneous manifestations, but it did not achieve the therapeutic goal. However, with the prolonged use of corticosteroids, the patient developed steroid-induced myopathy; so the dose had to be decreased to avoid worsening of muscle weakness.

The use of azathioprine and hydroxychloroquine successfully treated the patient’s muscular disease and caused the levels of CK and CKMB to normalize. However, the skin lesions persisted. Rituximab was studied previously in refractory skin manifestation of dermatomyositis with patients achieving remission with therapy; however, our patient’s skin lesions did not respond to rituximab [7].

IVIG was also assessed as a treatment modality for refractory cutaneous manifestation of dermatomyositis in other studies, showing an 83% rate of improvement in patients who had refractory cutaneous dermatomyositis, and an 81% rate of improvement in patients who had extra-cutaneous manifestations [8]. This is also the case in our patient, as he had significant improvement in his skin lesions upon initiation of treatment. Another benefit of IVIG is the cost-effectiveness of the therapy, compared to the clinical efficacy in the treatment outcomes. Adverse effects can occur with IVIG treatment but usually are mild such as headaches, fevers, chills, nausea, and vomiting. Some late adverse may occur, including acute kidney failure, pseudohyponatremia, thromboembolic events, and aseptic meningitis. However, appropriate hydration with a slow infusion rate helps avoid these complications, with monitoring of kidney functions and urine output [9].

Studies have also assessed the response of steroid-resistant dermatomyositis with elevated levels of creatine kinase to IVIG and found favorable outcomes, with improvement seen from the first treatment cycle as well [10]. Other studies have also assessed possible alternative treatment options such as Janus kinase inhibitors such as tofacitinib, which proved its efficacy against refractory cutaneous and extra-cutaneous manifestations of dermatomyositis [11]. Medications targeting interferon pathways have also been recently explored in patients with refractory cutaneous dermatomyositis, such as anifrolumab, which has shown to have good clinical outcomes [12].

## Conclusions

Treatment of dermatomyositis can be particularly challenging, as there are multiple manifestations of the disease not all of which can be responsive to the same line of treatment. Prolonged courses of corticosteroid use with patients with refractory disease also expose patients to the risk of steroid-induced myopathy, as observed in our patient. In this case report, the use of IVIG in such refractory cases was explored with good response; however, upon attempting to increase the interval between doses, there was a recurrence of the skin lesions. As such, for patients who have failed multiple lines of management, IVIG dosed at a four-week interval is an effective treatment regimen for refractory cases of dermatomyositis.
